# Anterior wall adenocarcinoma of bladder with similar clinicopathological and prognostic characteristics as common bladder carcinomas should not be treated as or classified into urachal adenocarcinomas

**DOI:** 10.1002/cam4.4053

**Published:** 2021-07-18

**Authors:** Yu‐Wen Zhou, Yi‐Xiu Long, Kun Song, Li‐Bo Liang, Ke Cheng, Ye Chen, Ji‐Yan Liu

**Affiliations:** ^1^ Department of Biotherapy Cancer Center West China Hospital of Sichuan University Chengdu China; ^2^ Department of Abdominal Oncology Mei Shan Cancer Hospital Mei Shan China; ^3^ Department of Abdominal Oncology Cancer Center West China Hospital of Sichuan University Chengdu China

**Keywords:** bladder adenocarcinoma, non‐urachal adenocarcinoma, primary tumor site, SEER, surgery, urachal adenocarcinoma

## Abstract

**Purpose:**

To discuss whether the dome or anterior wall of bladder adenocarcinoma (BAC) should be classified into urachal carcinoma (UrC) and the relationship of primary tumor location (PTL) as well as treatment with survival.

**Methods:**

Surveillance, Epidemiology, and End Results 18 database was examined for eligible patients from 1975 to 2016. Patients were classified into adenocarcinoma originating from the urachus (UAC), the dome (D‐BAC), the anterior wall (A‐BAC), and the other sites adenocarcinoma of the bladder (O‐BAC). The clinicopathological features, treatment, and survival were compared among the groups.

**Results:**

Comparable clinicopathologic features were obtained between UAC and D‐BAC, which were different from those of A‐BAC and O‐BAC; otherwise, the latter two had similar clinicopathologic features. Univariable and multivariable Cox regression analyses indicated that PTL was an independent predictor for survival. O‐BAC conferred the worst prognosis then followed by A‐BAC, D‐BAC, and UAC. For non‐metastatic UAC or D‐BAC, partial cystectomy (with an en bloc resection of the urachus and umbilicus) is optimal for survival. However, the worse survival of non‐metastatic D‐BAC (compared with UAC) suggested different modalities, maybe more intensive surgery approaches, should be considered for D‐BAC.

**Conclusion:**

This study illustrates that PTL of UAC and BAC was an independent predictor for survival. A‐BAC had comparable characters and prognosis with O‐BAC and should not be classified into and treated as UrC. For non‐metastatic disease, non‐metastatic D‐BAC may need more intensive modality.

## Graphical abstract

Anterior wall of bladder adenocarcinoma (A‐BAC) had comparable characters and prognosis with other sites of bladder adenocarcinoma, while dome adenocarcinoma of the bladder is similar to urachal adenocarcinoma arising from the urachus. A‐BAC should be classified into and treated as BAC, instead of urachal carcinoma.

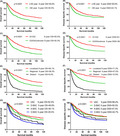

## 1 INTRODUCTION

Urachus, arising from the degeneration of allantois during embryogenesis, is a tubular structure that connects the urinary bladder to the umbilicus. When the physiological lumen obliteration fails, the urachal remnant, characterized by a tubular or cystic muscular structure lined by epithelium, persists in a minority of adults.^1^, ^2^ Neoplasms originating from the urachal remnant are also defined as urachal carcinoma (UrC). Because it is rare and sometimes tricky to differentiated from bladder adenocarcinoma (BAC), UrC is commonly accounted for bladder carcinoma (accounts for less than 1% of all bladder carcinoma).^1^, ^3^, ^4^ However, typical UrC demonstrates clinical and pathologic features that are different from those of bladder carcinomas.^5^, ^6^, ^7^, ^8^ Urachal adenocarcinoma (UAC) is the primary pathological type of UrC^9^, ^10^ and is less common than BAC.^11^

Due to its location relationship with the bladder, the diagnosis of UrC is usually confused, and its diagnosis and treatment also remain a challenge.^12^ Several stage classification genres of UrC have been proposed, but the most often applied are the Sheldon and Mayo Staging Systems,^13^ including the tumor situated in the bladder dome and/or anterior wall, whose diagnostic criteria are adopted and included in that of World Health Organization (WHO) (2016). The classification of the dome and anterior wall into the UrC might be based on the comprehension of the origin of the urachal ligament, but the rationality of classification is worth further discussion. Definitely, published studies have recently indicated significantly different clinical characteristics and prognoses between the UAC and BAC.^14^, ^15^ However, in these studies, the urachus and dome adenocarcinoma of the bladder were usually classified into UAC, showing marked differences in characteristics and prognosis from BAC. Otherwise, cancers from the anterior wall of the bladder were not clearly described.^14^, ^15^, ^16^ Up to now, no study has been reported to illustrate the discrepancy of the clinical characteristics and prognosis between the cancers of the dome, anterior wall of the bladder, and urachus separately. The aim of this study, based on the analysis of the clinicopathological features, treatment, and prognostic factors of primary tumor sites of UAC and BAC, is to furtherly discuss whether the dome or/and anterior wall adenocarcinoma of bladder should be classified into UrC.

## 2 MATERIALS AND METHODS

### 2.1 Patient selection

The Surveillance, Epidemiology, and End Results (SEER) 18 database, maintained by the National Cancer Institute (Bethesda, MD), was examined for all UAC and BAC patients from 1975 to 2016. The data used and analyzed in our study can be downloaded from the SEER (https://seer.cancer.gov/). Patients who meet the following selection criteria were reviewed and included in this study: (i) tumor located in the dome, anterior wall, other sites of the bladder as well as urachus (the third edition of International Statistical Classification of Disease for Oncology (ICD‐O‐3), primary site code C.67.0–C67.9); (ii) diagnosed from the January 1, 1975 to December 31, 2016; (iii) pathologically confirmed tumor; (iv) pathology encoded with adenocarcinoma (mucinous cells, signet ring cells, papillary cells, clear cells, mixed subtypes, intestinal type, and ordinary adenocarcinoma [not specified]). Patients without complete information, death within 30 days after follow‐up, and not first tumor were excluded from this study.

Demographics (gender, race, and age at diagnosis), as well as clinical parameters (tumor grade, SEER stage, histologic type, tumor‐related surgical methods, chemotherapy, and radiotherapy) of the patients with the diagnosis of urachal and BACs were extracted via the option of “case listing.” For analysis purposes, eligible patients were classified into adenocarcinomas arising from urachus (UAC), the dome (D‐BAC), the anterior wall (A‐BAC) of the bladder, and other sites of the BAC (O‐BAC). For D‐BAC and A‐BAC, they may include cancers originating from urachus or bladder. Complete resection of urachus and navel, as well as partial cystectomy, are currently considered as the standard surgical methods for UAC. However, given that no detailed information regarding the total urachus and navel resection is provided in the database of SEER, we speculated that this surgical approach was classified into partial cystectomy.

### 2.2 Statistical analysis

Data exported from the latest SEER*Stat 8.3.6 software version were stored in Microsoft Excel 2016 (Microsoft Corporation). Kaplan–Meier survival curves were utilized for both overall survival (OS) and disease‐specific survival (DSS). In our study, survival represents the date from cancer diagnosis to death. At the time of the last follow‐up, patients presumed alive were censored. Hazards ratios (HR) for the OS and DSS were calculated using the Cox Proportional Hazards Regression Model. Statistical analysis was conducted using the SPSS 25.0 software (SPSS Inc.). Statistical significance was defined as *p* values less than 0.05 in a two‐tailed test.

## 3 RESULTS

### 3.1 Screening patients

A total of 3779 cases pathologically confirmed UAC or BAC, diagnosed between 1975 and 2016, were identified; 457 cases lacked complete survival materials for analysis, and five cases died within 30 days after the follow‐up. Patients with not first tumor (*n* = 1314) were excluded from the present study. Ultimately, 2003 cases were included in this study. All patients were grouped by primary site, including 314 cases of UAC, 358 D‐BAC, 70 A‐BAC, and 1,261 O‐BAC. Demographic data for all eligible ones in this study are shown in Table 1.

**TABLE 1:** Baseline characteristics of eligible patients

	UAC *n* (%)	D‐BAC *n* (%)	A‐BAC *n* (%)	O‐BAC *n* (%)	*p*
Total	314	358	70	1261	
Gender					<0.001
Male	167 (53.2)	194 (54.2)	44 (62.9)	819 (64.9)	
Female	147 (46.8)	164 (45.8)	26 (37.1)	442 (35.1)	
Age categories					<0.001
<45	84 (26.8)	52 (14.5)	9 (12.9)	81 (6.4)	
45–60	118 (37.6)	124 (34.6)	17 (24.3)	329 (26.1)	
61–75	94 (29.9)	115 (32.1)	23 (32.9)	446 (35.4)	
>75	18 (5.7)	67 (18.7)	21 (30.0)	405 (32.1)	
Race					0.005
White	232 (73.9)	273 (76.3)	56 (80.0)	1000 (79.3)	
Black	35 (11.1)	47 (13.1)	9 (12.9)	205 (16.3)	
Other	47 (15.0)	38 (10.3)	5 (7.1)	56 (4.4)	
Grade					<0.001
G1	42 (13.4)	30 (8.4)	4 (5.7)	73 (5.8)	
G2	106 (33.8)	135 (37.7)	28 (40.0)	226 (17.9)	
G3	66 (21.0)	110 (30.7)	15 (21.4)	491 (38.9)	
G4	11 (3.5)	20 (5.6)	9 (12.9)	157 (12.5)	
Unknown	89 (28.3)	63 (17.6)	14 (20.0)	314 (24.9)	
Histology					<0.001
Adenocarcinoma NOS	122 (38.9)	189 (52.8)	41 (58.6)	707 (56.1)	
Intestinal type AC	3 (1.0)	4 (1.1)	1 (1.4)	3 (0.2)	
Mucinous AC	162 (51.6)	112 (31.3)	10 (14.3)	163 (12.9)	
Papillary AC	4 (1.3)	10 (2.8)	3 (4.3)	68 (5.4)	
Signet ring cell carcinoma	15 (4.8)	29 (8.1)	7 (10.0)	179 (14.2)	
Clear cell AC	2 (0.6)	3 (0.8)	3 (4.3)	76 (6.0)	
Mixed cell AC	6 (1.9)	11 (3.1)	5 (7.1)	65 (5.2)	
SEER stage					<0.001
Localized	510 (25.5)	49 (15.6)	69 (19.3)	17 (24.3)	
Regional	989 (49.4)	174 (55.4)	235 (65.6)	36 (51.4)	
Distant	415 (20.7)	83 (26.4)	46 (12.8)	16 (22.9)	
Unknown	89 (4.4)	8 (2.5)	8 (2.2)	1 (1.4)	

Abbreviations: A‐BAC, anterior wall of bladder adenocarcinoma; AC, adenocarcinoma; D‐BAC, doom adenocarcinoma of the bladder; O‐BAC, other sites of bladder adenocarcinoma; UAC, urachal adenocarcinoma arising from urachus.

### 3.2 Baseline characteristics

A higher proportion of males could be observed in each primary location subset. The percentages of males were comparable between A‐BAC (62.9%) and O‐BAC (64.9%) groups, which were slightly higher than those in UAC (53.2%) and D‐BAC (54.2%) groups. UAC or D‐BAC patients were significantly younger than A‐BAC or O‐BAC patients, with proportions of patients diagnosed under 60 years of age were 64.4% for UAC, 49.1% for D‐BAC, 37.2% for A‐BAC, and 32.5% for O‐BAC, respectively. Mucinous adenocarcinoma was the main pathological type of UAC, while the common adenocarcinoma was the primary pathological type of other tumors located in the dome, anterior, and other walls of the bladder. However, mucinous adenocarcinoma account for a significantly higher proportion in D‐BAC (31.3%) than that in A‐BAC (14.3%) and O‐BAC (12.9%), which showed that the pathological feature of D‐BAC was more inclined to that of UAC. Concerning grade and stage, no significant differences were observed among groups (Table 1.).

### 3.3 Survival analysis of all eligible patients

**FIGURE 1:**
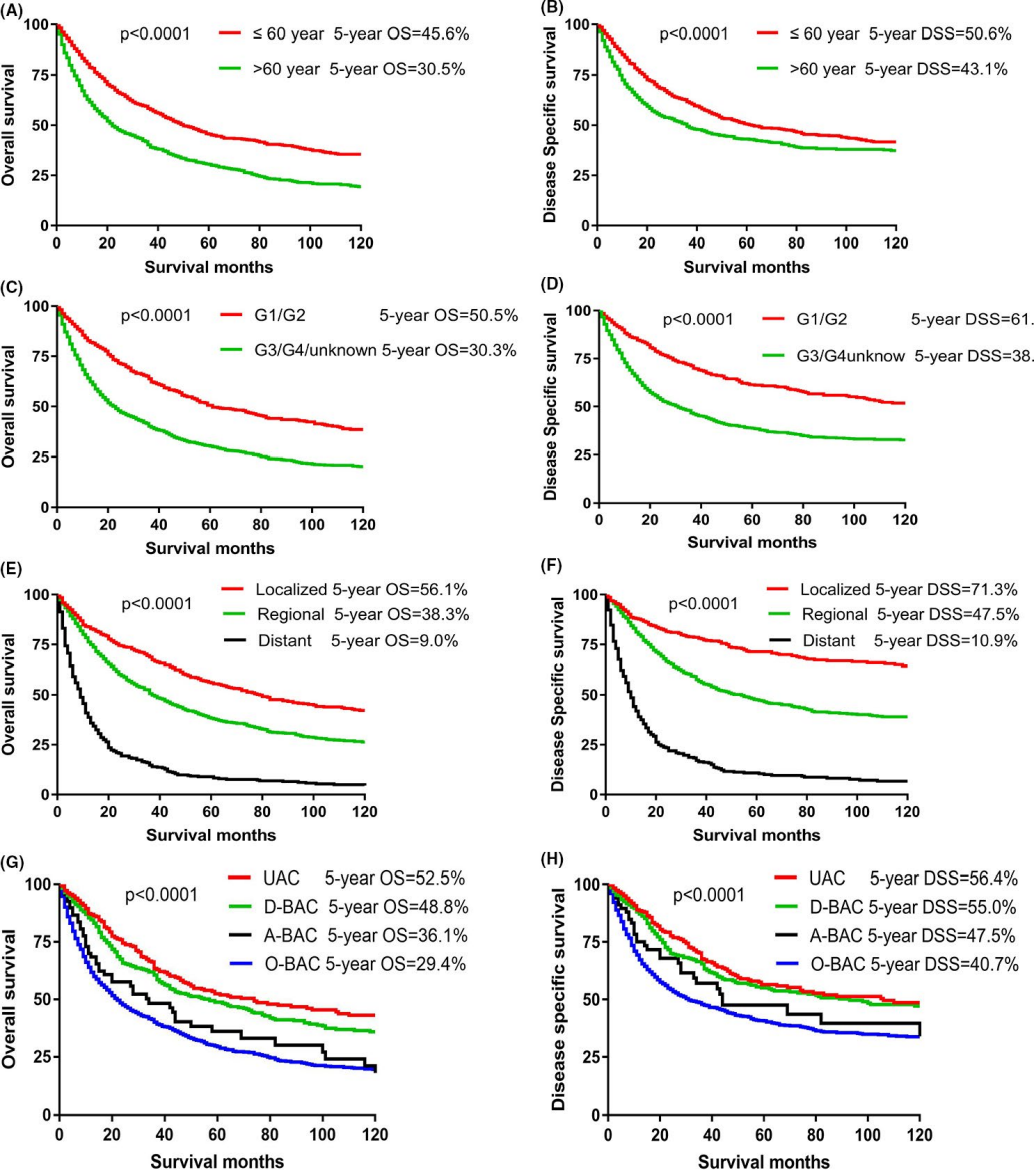
Kaplan–Meier curves of OS and DSS for all eligible patients. DSS, disease‐specific survival; OS, overall survival

To analyze the clinicopathological characteristics, particularly the impact of tumor sites of adenocarcinoma on survival, univariable and multivariable analyses were performed for all enrolled patients. The age ≤ 60, low tumor grade, mucinous adenocarcinoma, and early SEER stage have been demonstrated independent predictors of good prognosis for OS and DSS in both univariable and multivariable analyses (Table 2). Statistically significant variables for both 5‐year OS and DSS rate calculated by Kaplan–Meier analysis are shown in Figure 1. UAC generally had better survival than BAC (Figure 1g,h). Notably, as far as the impact of primary tumor sites on OS, UAC conferred the best prognosis, then followed by D‐BAC (HR = 1.260; 95% CI: 1.024–1.550; *p* = 0.029), A‐BAC (HR = 1.936; 95% CI: 1.409–2.661; *p* < 0.001) and finally O‐BAC (HR = 2.128; 95% CI: 1.791–2.529; *p* < 0.001), and the same trend was also obtained in the analysis for DSS (Table 2), which indicated that tumor locations were essential factors of prognosis. Meanwhile, a comparable 5‐year DSS rate was obtained in UAC (56.4%) and D‐BAC (55.0%) groups, and a similar 5‐year DSS rate was also observed in A‐BAC (47.5%) and O‐BAC (40.7%) groups (Figure 1). After excluding UAC and D‐BAC, A‐BAC has a similar prognosis as O‐BAC in both the univariable and multivariable analyses (Table S1).

**TABLE 2:** Univariable and Multivariable Cox analyses of determinants of OS and DSS for all eligible patients

	OS		DSS	
	HR (95% CI)	*p*‐value	HR (95% CI)	*p*‐value
*Univariable analysis*				
Gender				
Male	Reference		Reference	
Female	1.074 (0.963–1.197)	0.199	1.218 (1.075–1.379)	0.002
Age				
≤60 years	Reference		Reference	
>60 years	1.821 (1.627–2.039)	<0.001	1.324 (1.167–1.501)	<0.001
Race				
White	Reference		Reference	
Others	0.878 (0.769–1.002)	0.054	0.934 (0.804–1.084)	0.367
Grade				
Grade 1/2	Reference		Reference	
Grade 3/4/ unknown	1.788 (1.585–2.017)	<0.001	1.985 (1.718–2.293)	<0.001
Histology				
Mucinous AC	Reference		Reference	
Non‐mucinous AC	1.426 (1.248–1.629)	<0.001	1.335 (1.148–1.554)	<0.001
SEER stage				
Localized	Reference		Reference	
Regional	1.438 (1.257–1.646)	<0.001	2.053 (1.709–2.467)	<0.001
Distant	3.949 (3.379–4.615)	<0.001	6.777 (5.564–8.255)	<0.001
PTL				
Urachus	Reference		Reference	
Dome	1.260 (1.024–1.550)	0.029	1.143 (0.903–1.446)	0.265
Anterior wall	1.936 (1.409–2.661)	<0.001	1.439 (0.975–2.125)	0.067
Other positions	2.128 (1.791–2.529)	<0.001	1.827 (1.506–2.216)	<0.001
*Multivariable analysis*				
Gender				
Male	Reference		Reference	
Female	1.103 (0.988–1.231)	0.082	1.207 (1.062–1.372)	0.004
Age				
≤60 years	Reference		Reference	
>60 years	1.680 (1.496–1.887)	<0.001	1.278 (1.120–1.458)	<0.001
Grade				
Grade 1,2	Reference		Reference	
Grade 3,4/unknown	1.429 (1.261–1.618)	<0.001	1.601 (1.375–1.863)	<0.001
Histology				
Mucinous AC	Reference		Reference	
Non‐mucinous AC	1.229 (1.064–1.420)	0.005	1.209 (1.024–1.462)	0.025
SEER stage				
Localized	Reference		Reference	
Regional	1.755 (1.527–2.017)	<0.001	2.364 (1.960–2.852)	<0.001
Distant	4.954 (4.214–5.825)	<0.001	7.978 (6.511–9.775)	<0.001
PTL				
Urachus	Reference		Reference	
Dome	1.411 (1.139–1.848)	0.002	1.444 (1.131–1.843)	0.003
Anterior wall	1.812 (1.306–2.514)	<0.001	1.612 (1.081–2.404)	0.019
Other positions	2.059 (1.707–2.485)	<0.001	2.024 (1.639–2.498)	<0.001

Abbreviations: AC, adenocarcinoma; DSS, disease‐specific survival; OS, overall survival; PTL, primary tumor locations.

### 3.4 Analyses in non‐metastatic patients

**FIGURE 2:**
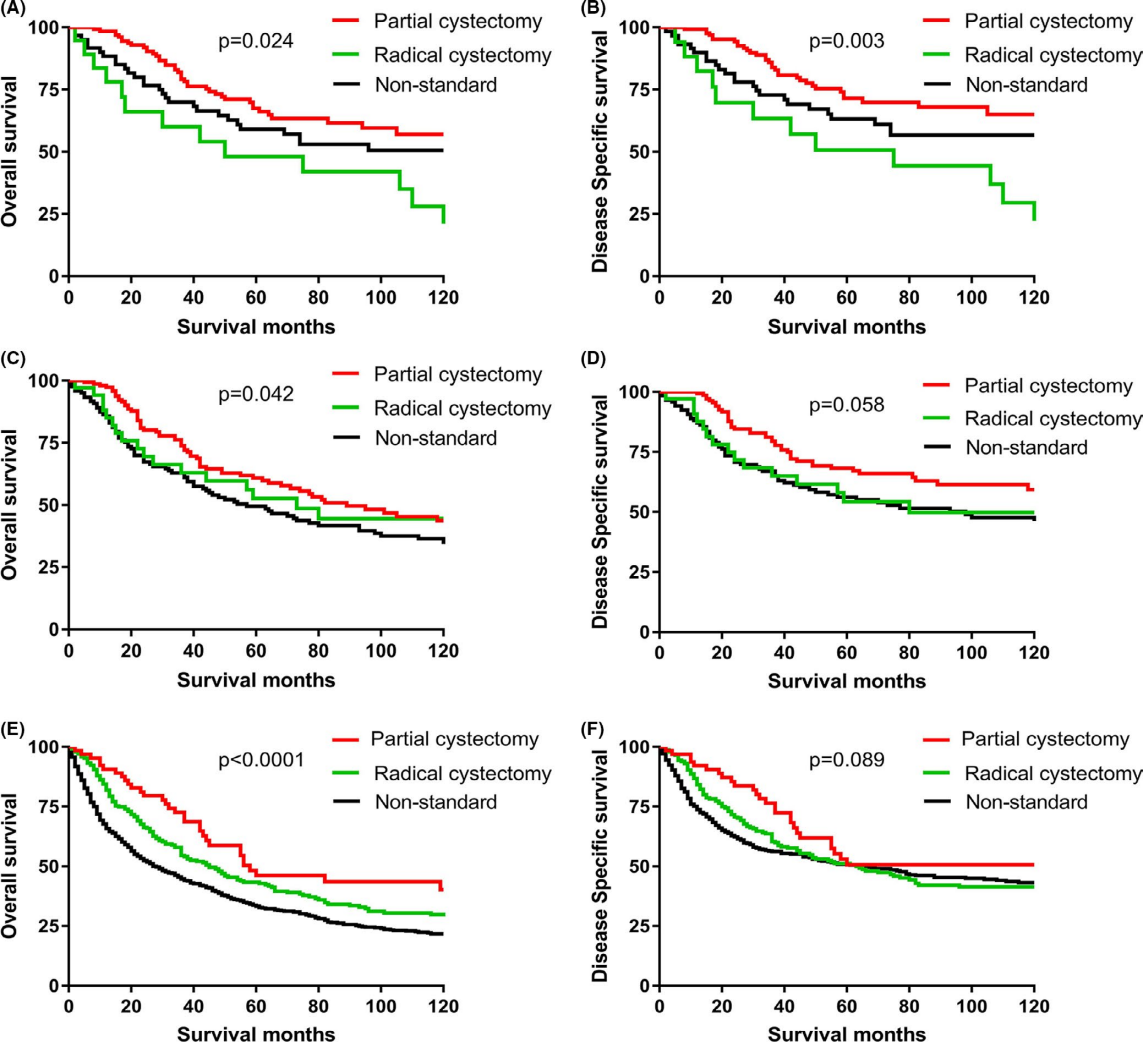
Kaplan–Meier curves of the approach of surgery on OS and DSS for localized diseases of UAC (A, B), D‐BAC (C, D), and A‐BAC/O‐BAC (e and f). DSS, disease‐specific survival; OS, overall survival

To further understand the impact of treatment on survival of non‐metastatic UAC and BAC, we extracted the data from localized and regional diseases and divided the eligible patients into UAC, D‐BAC, and BAC (containing A‐BAC and O‐BAC due to the relatively fewer cases of A‐BAC and its similar characteristics to O‐BAC). The indicators for OS and DSS were analyzed in the univariable and multivariable Cox regression analyses. The grade and SEER stage were also demonstrated as independent predictors of prognosis for some OS and DSS. Importantly, partial cystectomy seems to be shown the optimal treatment of surgery in patients with UAC or D‐BAC, both radical cystectomy and non‐standard surgery approach (other approaches of non‐radical cystectomy or absence of cancer‐related surgery) would significantly increase the risk of death. Interestingly, although partial cystectomy illustrates numerically benefit for D‐BAC, the advantage is not as remarkable as that for UAC. Otherwise, the death risk of radical cystectomy is relatively lower in D‐BAC than in UAC (Figure 2.) No groups of non‐metastatic patients were found to benefit from chemotherapy or radiotherapy (Table 3).

**TABLE 3:** Univariable and multivariable Cox analyses of determinants of OS and DSS for non‐metastatic patients with UAC, D‐BAC, and O‐BAC/A‐BAC

	UAC (*n* = 223)				D‐BAC (*n* = 304)				O‐BAC (*n* = 53)/A‐BAC (*n* = 919)			
	OS		DSS		OS		DSS		OS		DSS	
	HR (95% CI)	*p*	HR (95% CI)	*p*	HR (95% CI)	*P*	HR (95% CI)	*p*	HR (95% CI)	*p*	HR (95% CI)	*p*
Univariable sex												
Male	Reference		Reference		Reference		Reference		Reference		Reference	
Female	1.053 (0.696–1.591)	0.808	1.421 (0.881–2.292)	0.149	1.068 (0.795–1.434)	0.663	1.421 (0.994–2.030)	0.054	1.279 (1.100–1.486)	0.001	1.473 (1.222–1.775)	<0.001
Age												
≤60 years	Reference		Reference		Reference		Reference		Reference		Reference	
>60 years	1.474 (0.960–2.262)	0.076	0.837 (0.488–1.435)	0.517	1.611 (1.196–2.168)	0.002	1.191 (0.834–1.700)	0.336	1.964 (1.660–2.322)	<0.001	1.361 (1.116–1.659)	0.002
Race												
White	Reference		Reference		Reference		Reference		Reference		Reference	
Others	1.162 (0.728–1.852)	0.529	1.147 (0.669–1.965)	0.619	0.943 (0.666–1.334)	0.739	0.935 (0.616–1.420)	0.753	0.823 (0.681–0.994)	0.043	0.974 (0.777–1.221)	0.821
Grade												
1/2	Reference		Reference		Reference		Reference		Reference		Reference	
3/4/unknown	1.489 (0.983–2.257)	0.060	1.941 (1.186–3.178)	0.008	1.483 (1.100–2.001)	0.010	1.520 (1.058–2.183)	0.023	1.478 (1.249–1.750)	<0.001	1.664 (1.334–2.077)	<0.001
Histology												
MAC	Reference		Reference		Reference		Reference		Reference		Reference	
NMAC	1.224 (0.811–1.849)	0.335	1.117 (0.694–1.798)	0.647	0.922 (0.676–1.256)	0.606	1.004 (0.686–1.470)	0.982	1.092 (0879–1.356)	0.427	0.916 (0.711–1.179)	0.495
SEER stage												
Localized	Reference		Reference		Reference		Reference		Reference		Reference	
Regional	1.288 (0.759–2.185)	0.348	1.359 (0.728–2.537)	0.335	1.766 (1.215–2.568)	0.002	2.995 (1.712–5.240)	<0.001	1.760 (1.508–2.054)	<0.001	2.422 (1.967–2.982)	<0.001
Surgery												
RC	2.253 (1.208–4.205)	0.011	2.954 (1.518–5.748)	0.001	1.295 (0.786–2.134)	0.309	1.623 (0.932–2.828)	0.087	Reference		Reference	
PC	Reference		Reference		Reference		Reference		0.669 (0.463–0.968)	0.033	0.708 (0.463–1.082)	0.111
NS/unknown	1.438 (0.911–2.269)	0.119	1.509 (0.887–2.568)	0.129	1.497 (1.089–2.059)	0.013	1.528 (1.038–2.248)	0.032	1.339 (1.124–1.595)	0.001	1.090 (0.883–1.345)	0.425
Chemotherapy												
Yes	Reference		Reference		Reference		Reference		Reference		Reference	
No	0.882 (0.523–1.515)	0.648	0.705 (0.397–1.251)	0.232	0.703 (0.484–1.021)	0.064	0.599 (0.394–0.910)	0.016	0.793 (0.660–0.953)	0.013	0.690 (0.556–0.857)	0.001
Radiotherapy												
Yes	Reference		Reference		Reference		Reference		Reference		Reference	
No	0.462 (0.223–0.957)	0.038	0.396 (0.181–0.869)	0.021	0.649 (0.403–1.046)	0.076	0.626 (0.359–1.093)	0.099	0.680 (0.550–0.839)	<0.001	0.643 (0.498–0.829)	0.001
Multivariable sex												
Male	Reference		Reference		Reference		Reference		Reference		Reference	
Female	1.025 (0.671–1.566)	0.910	1.305 (0.799–2.131)	0.287	1.014 (0.750–1.370)	0.929	1.292 (0.897–1.859)	0.168	1.192 (1.021–1.391)	0.026	1.380 (1.157–1.647)	<0.001
Age												
≤60 years	Reference		Reference		Reference		Reference		Reference		Reference	
>60 years	1.816 (1.149–2.871)	0.011	1.043 (0.587–1.854)	0.885	1.595 (1.179–2.159)	0.002	1.210 (0.843–1.735)	0.301	1.804 (1.512–2.154)	<0.001	1.304 (1.057–1.609)	0.013
Race												
White	Reference		Reference		Reference		Reference		Reference		Reference	
Others	1.131 (0.701–1.823)	0.614	0.952 (0.544–1.665)	0.862	1.072 (0.753–1.528)	0.699	1.080 (0.707–1.652)	0.721	0.855 (0.702–1.041)	0.119	0.933 (0.738–1.181)	0.565
Grade												
1,2	Reference		Reference		Reference		Reference		Reference		Reference	
3,4/known	1.446 (0.950–2.202)	0.085	1.964 (1.191–3.239)	0.008	1.377 (1.012–1.875)	0.042	1.438 (0.992–2.084)	0.055	1.248 (1.047–1.488)	0.014	1.310 (1.082–1.585)	0.006
Histology												
MAC	Reference		Reference		Reference		Reference		Reference		Reference	
NMAC	1.212 (0.790–1.861)	0.378	1.071 (0.854–1.755)	0.785	1.021 (0.737–1.415)	0.901	1.012 (0.678–1.510)	0.953	0.925 (0.738–1.158)	0.495	0.869 (0.667–1.132)	0.297
SEER stage												
Localized	Reference		Reference		Reference		Reference		Reference		Reference	
Regional	1.424 (0.808–2.508)	0.222	1.295 (0.657–2.552)	0.455	2.230 (1.474–3.373)	<0.001	3.721 (2.040–6.789)	<0.001	2.230 (1.867–2.662)	<0.001	2.694 (2.135–3.400)	<0.001
Surgery												
RC	3.060 (1.538–6.088)	0.001	3.379 (1.618–7.055)	0.001	1.102 (0.660–1.842)	0.710	1.371 (0.774–2.429)	0.280	Reference		Reference	
PC	Reference		Reference		Reference		Reference		0.820 (0.562–1.196)	0.303	0.871 (0.564–1.345)	0.534
NS/unknown	1.570 (0.970–2.542)	0.066	1.676 (0.959–2.930)	0.707	1.747 (1.240–2.461)	0.010	2.058 (1.368–3.096)	0.001	1.786 (1.476–2.161)	<0.001	1.629 (1.300–2.041)	<0.001
Chemotherapy												
Yes	Reference		Reference		Reference		Reference		Reference		Reference	
No	1.298 (0.715–2.356)	0.391	1.091 (0.580–2.054)	0.787	0.765 (0.502–1.166)	0.213	0.691 (0.433–1.103)	0.121	0.937 (0.769–1.141)	0.516	0.902 (0.716–1.137)	0.382
Radiotherapy												
Yes	Reference		Reference		Reference		Reference		Reference		Reference	
No	0.319 (0142–0.714)	0.005	0.327 (0.136–0.783)	0.012	0.921 (0.536–1.583)	0.765	0.988 (0.527–1.854)	0.971	0.919 (0.738–1.146)	0.454	0.900 (0.690–1.175)	0.440

Note

Non‐standard represents other approach of non‐radical cystectomy or absence of cancer‐related surgery.

Abbreviations: A‐BAC, anterior wall of bladder adenocarcinoma; D‐BAC, dome adenocarcinoma of the bladder; MAC, mucinous adenocarcinoma; NS, non‐standard; O‐BAC, other sites of bladder adenocarcinoma; PC, partial cystectomy; RC, radical cystectomy; UAC, urachal adenocarcinoma arising from the urachus.

### 3.5 Analyses in metastatic patients

As for the impact of treatment on metastatic diseases, palliative surgery seemed to decrease the risk of death in patients with UAC and BAC in both the univariable and multivariable analyses. Moreover, patients with metastatic A‐BAC or O‐BAC could significantly benefit from systemic chemotherapy, but radiotherapy did not take any survival benefits to metastatic patients (Table 4).

**TABLE 4:** Univariable and multivariable Cox analyses of determinants of OS and DSS for metastatic patients with UAC, D‐BAC, and O‐BAC/A‐BAC

	UAC (*n* = 83)				D‐BAC (*n* = 46)				O‐BAC/A‐BAC (*n* = 286)			
	OS		DSS		OS		DSS		OS		DSS	
	HR (95% CI)	*p*	HR (95% CI)	*p*	HR (95% CI)	*p*	HR (95% CI)	*p*	HR (95% CI)	*p*	HR (95% CI)	*p*
Univariable sex												
Male	Reference		Reference		Reference		Reference		Reference		Reference	
Female	1.034 (0.616–1.736)	0.900	1.035 (0.602–1.778)	0.902	1.509 (0.807–2.823)	0.198	1.486 (0.768–2.876)	0.240	0.984 (0.764–1.267)	0.899	0.968 (0.743–1.262)	0.812
Age												
≤60 years	Reference		Reference		Reference		Reference		Reference		Reference	
>60 years	1.096 (0.639–1.883)	0.738	1.159 (0.662–2.029)	0.607	0.946 (0.499–1.795)	0.865	0.900 (0.461–1.756)	0.757	1.368 (1.061–1.762)	0.016	1.328 (1.020–1.730)	0.035
Race												
White	Reference		Reference		Reference		Reference		Reference		Reference	
Others	1.516 (0.893–2.576)	0.124	1.387 (0.791–2.430)	0.253	1.144 (0.562–2.329)	0.710	1.329 (0.641–2.752)	0.444	0.868 (0.627–1.201)	0.393	0.884 (0.631–1.238)	0.472
Grade												
½	Reference		Reference		Reference		Reference		Reference		Reference	
¾	2.166 (1.218–3.850)	0.008	2.314 (1.258–4.260)	0.007	1.221 (0.636–2.342)	0.548	1.492 (0.734–3.031)	0.269	1.725 (1.207–2.467)	0.003	1.594 (1.107–2.294)	0.012
Histology												
MAC	Reference		Reference		Reference		Reference		Reference		Reference	
NMAC	2.022 (1.203–3.398)	0.008	1.941 (1.127–3.343)	0.017	1.056 (0.530–2.104)	0.877	1.398 (0.640–3.053)	0.401	1.631 (1.103–2.412)	0.014	1.689 (1.112–2.565)	0.014
Palliative surgery												
Yes	Reference		Reference		Reference		Reference		Reference		Reference	
No	2.192 (1.221–3.935)	0.009	2.285 (1.245–4.199)	0.008	2.576 (0.871–7.623)	0.087	2.035 (0.595–6.956)	0.257	1.454 (1.133–1.867)	0.003	1.458 (1.124–1.895)	0.005
Chemotherapy												
Yes	Reference		Reference		Reference		Reference		Reference		Reference	
No	1.037 (0.607–1.772)	0.894	1.096 (0.629–1.910)	0.746	1.509 (0.814–2.795)	0.191	1.447 (0.756–2.769)	0.265	1.593 (1.250–2.031)	<0.001	1.556 (1.207–2.007)	0.001
Radiotherapy												
Yes	Reference		Reference		Reference		Reference		Reference		Reference	
No	1.008 (0.495–2.054)	0.982	0.915 (0.446–1.875)	0.808	0.892 (0.423–1.882)	0.765	0.901 (0.409–1.984)	0.796	1.351 (0.984–1.855)	0.063	1.449 (1.031–2.036)	0.033
Multivariable sex												
Male	Reference		Reference		Reference		Reference		Reference		Reference	
Female	1.415 (0.777–2.575)	0.256	1.492 (0.794–2.802)	0.214	1.392 (0.689–2.810)	0.356	1.446 (0.696–3.003)	0.323	1.044 (0.806–1.353)	0.745	1.018 (0.776–1.336)	0.896
Age												
≤60 years	Reference		Reference		Reference		Reference		Reference		Reference	
>60 years	1.292 (0.735–2.271)	0.377	1.366 (0.758–2.461)	0.289	0.631 (0.265–1.503)	0.298	0.533 (0.218–1.300)	0.167	1.108 (0.851–1.441)	0.447	1.088 (0.827–1.432)	0.548
Race												
White	Reference		Reference		Reference		Reference		Reference		Reference	
Others	1.421 (0.813–2.484)	0.218	1.300 (0.719–2.348)	0.385	1.041 (0.472–2.297)	0.920	1.168 (0.520–2.628)	0.707	0.963 (0.687–1.350)	0.829	0.976 (0.689–1.386)	0.894
Grade												
½	Reference		Reference		Reference		Reference		Reference		Reference	
¾	1.943 (1.027–3.676)	0.041	2.139 (1.093–4.185)	0.026	1.413 (0.687–2.908)	0.348	1.713 (0.776–3.783)	0.183	2.009 (1.375–2.935)	<0.001	1.840 (1.252–2.703)	0.002
Histology												
MAC	Reference		Reference		Reference		Reference		Reference		Reference	
NMAC	1.627 (0.938–2.821)	0.083	1.543 (0.868–2.741)	0.140	1.280 (0.556–2.948)	0.562	1.934 (0.766–4.884)	0.163	1.821 (1.198–2.768)	0.005	1.832 (1.176–2.855)	0.007
Palliative surgery												
Yes	Reference		Reference		Reference		Reference		Reference		Reference	
No	2.116 (1.061–4.221)	0.033	2.356 (1.138–4.878)	0.021	2.363 (0.694–8.050)	0.169	1.881 (0.482–7.341)	0.363	1.361 (1.038–1.785)	0.026	1.336 (1.008–1.770)	0.044
Chemotherapy												
Yes	Reference		Reference		Reference		Reference		Reference		Reference	
No	1.046 (0.580–1.886)	0.881	1.052 (0.571–1.939)	0.871	1.614 (0.776–3.356)	0.200	1.723 (0.803–3.698)	0.163	1.913 (1.482–2.470)	<0.001	1.842 (1410–2.407)	<0.001
Radiotherapy												
Yes	Reference		Reference		Reference		Reference		Reference		Reference	
No	0.728 (0.340–1.561)	0.415	0.614 (0.283–1.332)	0.217	0.763 (0.325–1.789)	0.534	0.746 (0.310–1.791)	0.511	1.179 (0.834–1.665)	0.351	1.262 (0.874–1.823)	0.214

Abbreviations: A‐BAC, anterior wall of bladder adenocarcinoma; AC, adenocarcinoma; D‐BAC, dome adenocarcinoma of the bladder; O‐BAC, other sites of bladder adenocarcinoma; UAC, urachal adenocarcinoma arising from the urachus.

## 4 DISCUSSION

The UrC is a rare and malignant tumor with limited evidence to guide clinicians in its diagnosis and treatment. Although several small sample retrospective studies have recently been published.^9^, ^17^ Due to its rarity, treatment modalities and prognosis of UrC remain unclear, and prospective trials are still lacking. In accordance with the conclusion of previous studies on UAC, our study demonstrates that the age ≤ 60, low tumor grade, mucinous adenocarcinoma, early tumor stage, and primary tumor site are independent predictors of good prognosis and are also helpful to distinguish UAC from the BAC.^14^, ^16^, ^18^, ^19^ In addition, we also demonstrate that UAC is a fundamentally different disease from BAC and generally has better survival than BAC.

The definition of UrC generally includes carcinomas from the urachus, the dome, and the anterior wall of the bladder. However, a recent study performing the SEER database has found that UAC and BAC seemed to have discrepant outcomes. In these studies, neoplasms located in urachus or/and dome were commonly classified as UAC and showed a relatively better outcome than those in other sites of bladder^16^; otherwise, the A‐BAC was not specially classified. In the current study, we separated UAC, D‐BAC, A‐BAC from all eligible patients and compared the clinicopathologic characteristics and survival of different groups with BAC. This study, for the first time, from the perspective of baseline characteristics, such as gender, age, race, and grading, illustrates that A‐BAC has similar characters to O‐BAC, while D‐BAC is closer to UAC. Moreover, A‐BAC and O‐BAC have similar survival, while D‐BAC has a closer prognosis to UAC. Therefore, we agree that it is reasonable to classify UAC and D‐BAC as UrC, while it should be debated to categorize A‐BAC into UrC.

Currently, no standard treatment guideline is available for urachal malignant neoplasms. Treatment may be different for localized or metastatic UAC. For localized patients, the backbone therapy is surgery. It is very important for clinicians to differentiate UAC from BAC, as they may require different therapeutic strategies. The approach of surgery for UAC is partial cystectomy with complete resection of the mid‐umbilical ligament to the umbilicus.^6^ In contrast, BAC is commonly referred to as radical cystectomy for bladder urothelial carcinoma. In the current study, partial cystectomy is confirmed as the optimal treatment of surgery in patients with UAC or D‐BAC, while radical cystectomy would increase the risk of death (Figure 2). This result supports D‐BAC classification into UAC, and patients with D‐BAC or UAC should receive a similar surgery approach. Interestingly, we noticed that the prognosis of D‐BAC is worse than that of UAC in this study (Table 2). Considering the clinicopathological characteristics of D‐BAC are similar to those of UAC, we speculate that the difference of survival may result from the surgery approaches; that is, non‐metastatic D‐BAC gained a less advantage from partial cystectomy than non‐metastatic UAC. Our data also suggest that the relative risk of death for D‐BAC is lower than for UAC in patients who receive radical cystectomy. We suppose partial cystectomy may be insufficient to remove lesions in D‐BAC, or en bloc resection of the urachus and umbilicus plus radical cystectomy is worthy of being examined.

Apart from the primary tumor location, the classification of WHO (2016) for UrC cover the following criterion: predominant invasion of muscular or deeper tissues with sharp demarcation between tumor and surface bladder urothelium; surface urothelium is free of glandular or polypoid proliferation (i.e., invasion is from outside in); no carcinoma in situ or glandular metaplasia other than (possibly) cystitis glandular is present; the presence of urachal remnants is helpful but not always identifiable; no primary adenocarcinoma elsewhere. The revised classification seems reasonable; however, it is difficult for pathologists and clinicians to carry it out. For example, it is hard to differentiate the mucosal boundary from tumor invasion of the urothelium. Other criteria, such as glandular or polypoid proliferation, carcinoma in situ or glandular metaplasia, etc., should not be absolute factors for eliminating UrC.^13^ Moreover, the current WHO classification system hardly provides a reference for treatment options and prognosis assessment. Otherwise, different primary tumor sites, in spite of their origination from either urachus or bladder, do have an impact on the selection of treatment (especially on surgery approach) and prognosis. For example, even though some A‐BAC should be diagnosed as UrC according to WHO classification, the surgery approach should be taken as BAC. D‐BAC has similar clinicopathologic features and survival with UAC; thus, it is reasonable to be diagnosed as UrC; however, the surgical approach may need to be strengthened being non‐metastatic D‐BAC more likely to invade the bladder. Our introduction of en bloc resection of the urachus and umbilicus plus radical cystectomy is a reasonable theoretical recommendation but without sufficient evidence to support it. Prospective international collaborations or adequately powered trials may be needed to clarify it.

Several potential limitations are included in this study: a retrospective analysis based on the SEER database, whose parameters are assumed to have been coded and diagnosed accurately, but errors owing to oncologists may distort results; moreover, restricted descriptions of surgery approaches in the SEER database could restrain the ability of authors from differentiating partial cystectomy accurately, partial cystectomy may include en bloc resection of the urachus and umbilicus plus partial bladder resection and partial bladder resection alone. Theoretically, the inclusion of the latter may reduce the survival advantage of the former for UAC patients, contributing to the interference of prognostic analysis.

## 5 CONCLUSION

This study illustrates that the D‐BAC is similar to UAC, while A‐BAC is closer to O‐BAC in terms of clinicopathological features, treatment, and prognostic risk. A‐BAC should not be classified into and treated as UrC. Partial cystectomy (with an en bloc resection of the urachus and umbilicus) could be the prevailing treatment for patients with nonmetastatic UAC or D‐BAC but may not be sufficient for D‐BAC. Metastatic patients with A‐BAC or O‐BAC can significantly benefit from systemic chemotherapy.

## CONFLICT OF INTEREST

The authors declare no conflict of interest.

## Supporting information

Table S1

## Data Availability

The data that support the findings of this study are available from the SEER 18 database. If you need, the data used and analyzed in our study can be downloaded from SEER (https://seer.cancer.gov/). Another way is to contact our corresponding author.
