# Supplementary material for: Anterior wall adenocarcinoma of bladder with similar clinicopathological and prognostic characteristics as common bladder carcinomas should not be treated as or classified into urachal adenocarcinomas
Source: Cancer Med. 2021 Jul 18;10(16):5415–28. doi: 10.1002/cam4.4053 (PMC8366075; doi:10.1002/cam4.4053)
Supplement: Supplementary file 1 — Table S1 [file CAM4-10-5415-s001.docx]

**Supplementary Table 1.** Univariable and Multivariable Cox analysis of determinants of OS and DSS for all patients with A-BAC and O-BAC.

|  | **OS** | | **DSS** | |
| --- | --- | --- | --- | --- |
| **Univariable analysis** | **HR（95%CI）** | ***p-*value** | **HR（95%CI）** | ***p*-value** |
| **Gender** |  |  |  |  |
| Male | Reference |  | Reference |  |
| Female | 1.219(1.071-1.387) | 0.003 | 1.314(1.129-1.530) | <0.001 |
| **Age** |  |  |  |  |
| ≤ 60 year | Reference |  | Reference |  |
| ＞60 year | 1.685(1.466-1.937) | <0.001 | 1.257(1.073-1.472) | 0.005 |
| **Race** |  |  |  |  |
| White | Reference |  | Reference |  |
| Others | 0.816(0.693-0.960) | 0.014 | 0.915(0.759-1.104) | 0.354 |
| **Grade** |  |  |  |  |
| Grade 1/2 | Reference |  | Reference |  |
| Grade 3/4/ unknown | 1.609(1.384-1.871) | <0.001 | 1.796(1.488-2.467) | <0.001 |
| **Histology** |  |  |  |  |
| Mucinous AC | Reference |  | Reference |  |
| Non-mucinous AC | 1.195(0.989-1.444) | 0.085 | 1.076(0.867-1.334) | 0.508 |
| **SEER Stage** |  |  |  |  |
| Localized | Reference |  | Reference |  |
| Regional | 1.724(1.479-2.010) | <0.001 | 2.396(1.947-2.949) | <0.001 |
| Distant | 4.359(3.643-5.216) | <0.001 | 7.322(5.851-9.162) | <0.001 |
| **PTL** |  |  |  |  |
| Anterior wall | Reference |  | Reference |  |
| Other positions | 1.104(0.832-1.463) | 0.494 | 1.272(0.892-1.814) | 0.184 |
| **Multivariable analysis** |  |  |  |  |
| **Gender** |  |  |  |  |
| Male | Reference |  | Reference |  |
| Female | 1.112(0.976-1.267) | 0.111 | 1.178(1.011-1.374) | 0.036 |
| **Age** |  |  |  |  |
| ≤ 60 year | Reference |  | Reference |  |
| ＞60 year | 1.763(1.529-2.033) | <0.001 | 1.347(1.146-1.582) | <0.001 |
| **Grade** |  |  |  |  |
| Grade 1,2 | Reference |  | Reference |  |
| Grade 3,4/unknown | 1.334(1.144-1.557) | <0.001 | 1.480(1.221-1.795) | <0.001 |
| **Histology** |  |  |  |  |
| Mucinous AC | Reference |  | Reference |  |
| Non-mucinous AC | 1.214(1.001-1.472) | 0.049 | 1.153(0.926-1.436) | 0.202 |
| **SEER Stage** |  |  |  |  |
| Localized | Reference |  | Reference |  |
| Regional | 1.802(1.514-2.108) | <0.001 | 2.398(1.942-2.962) | <0.001 |
| Distant | 4.603(3.830-5.533) | <0.001 | 7.304(5.811-9.181) | <0.001 |
| **PTL** |  |  |  |  |
| Anterior wall | Reference |  | Reference |  |
| Other positions | 1.157(0.871-1.538) | 0.314 | 1.276(0.892-1.827) | 0.183 |

**Abbreviation OS**: overall survival; **DSS:** disease-specific survival; **AC:** adenocarcinoma; **PTL:** primary tumor locations.
